# MAG induces apoptosis in cerebellar granule neurons through p75^NTR^ demarcating granule layer/white matter boundary

**DOI:** 10.1038/s41419-019-1970-x

**Published:** 2019-09-30

**Authors:** Diana Fernández-Suárez, Favio A. Krapacher, Annika Andersson, Carlos F. Ibáñez, Lilian Kisiswa

**Affiliations:** 1 Department of Neuroscience, Karolinska Institute, S-17177 Stockholm, Sweden; 2 Department of Physiology, National University of Singapore, Singapore, 117597 Singapore; 3 Life Sciences Institute, National University of Singapore, Singapore, 117456 Singapore

**Keywords:** Apoptosis, Cell death in the nervous system, Molecular neuroscience

## Abstract

MAG (Myelin-associated glycoprotein) is a type I transmembrane glycoprotein expressed by Schwann cells and oligodendrocytes, that has been implicated in the control of axonal growth in many neuronal populations including cerebellar granule neurons (CGNs). However, it is unclear whether MAG has other functions in central nervous system, in particular, in cerebellar development and patterning. We find that MAG expression in the cerebellum is compartmentalised resulting in increased MAG protein levels in the cerebellar white matter. MAG induces apoptosis in developing CGNs through p75^NTR^ signalling. Deletion of p75^NTR^ in vivo reduced the number of apoptotic neurons in cerebellar white matter during development leading to reduction in the size of white matter in the adulthood. Furthermore, we show that MAG impairs CGNs neurite outgrowth as consequence of MAG-induced apoptosis in CGNs. Mechanistically, we find that MAG/NgR1-induced cell death is dependent of p75^NTR^-mediated activation of JNK/cell death signalling pathway. Together, these findings identify the mechanisms by which MAG induces CGNs apoptotic activity, a crucial event that facilitates cerebellar layer refinement during development.

## Introduction

The cerebellum is one of the most architecturally elaborated regions in the nervous system (NS). The fundamental determinant of cerebellar morphology is the correct allocation of different cell types in each specific territory. However, the molecular mechanisms governing the establishment and maintenance of the boundaries defining the different cerebellar regions are not fully understood. Cerebellar granule neurons (CGNs) start developing postnatally, forming a transitional cerebellar external granule layer (EGL), where the cells proliferate and then migrate to the internal granule layer (IGL)^1^. Although, molecules promoting CGNs migration have been identified^1^, it remains unclear what factors prevent CGNs migration beyond the granule layer (GL).

Myelin-associated glycoprotein (MAG) is a type I transmembrane protein expressed by myelinating glia, Schwann cells and oligodendrocytes of the peripheral and central nervous system (PNS and CNS, respectively), being preferentially enriched on the periaxonal layer of myelinated axons^2–6^. MAG functions as a bimodal factor, promoting axonal growth in embryonic neurons while inhibiting axonal growth in adult neurons, specifically in dorsal root ganglion neurons (DRG), retinal ganglion cells (RCG), spinal cord motor neurons, hippocampal neurons (HCN) superior cervical ganglion (SCG) neurons and CGNs^5–10^. MAG signals through several receptors including Nogo receptors 1 and 2 (NgR1 and NgR2)^11–13^. In the NS, MAG’s inhibitory effect on axonal growth requires the p75 neurotrophin receptor (p75^NTR^) as a co-receptor with NgR1, but not NgR2^8,13–16^.

p75^NTR^ activates several signalling pathways including NFkB^17–20^, JNK/cell death^21,22^ and RhoGDI/RhoA/ROCK^9,14,23–25^ depending on the availability of different ligands and adaptor proteins^20^. Through the p75^NTR^/NgR1 receptor complex, MAG activates the RhoGDI/RhoA/ROCK signalling pathway by recruiting the adaptor protein RhoGDI to the p75^NTR^ death domain, leading to growth cone collapse and axonal growth retardation^9,14,23–25^. Recently, one study showed that MAG/p75^NTR^ signalling impairs migration of Schwan cells and induces cell death in vitro and blockage of this pathway increases migration and survival of Schwan cells in demyelinating adult CNS^26^. We therefore ask whether MAG may prevent CGNs migration beyond the GL and into the white matter (WM).

In this study we examined the effect of MAG in developing CGNs and its contribution to defining the cerebellar GL/WM boundary. We found that MAG facilitates CGNs apoptotic activity both in vitro and in vivo, contributing to cerebellar layer refinement and to the maintenance of properly defined and functioning layers in adult cerebellum.

## Results

### MAG, its receptor (NgR1) and co-receptor (p75^NTR^) are expressed in the cerebellum

**Fig. 1:**
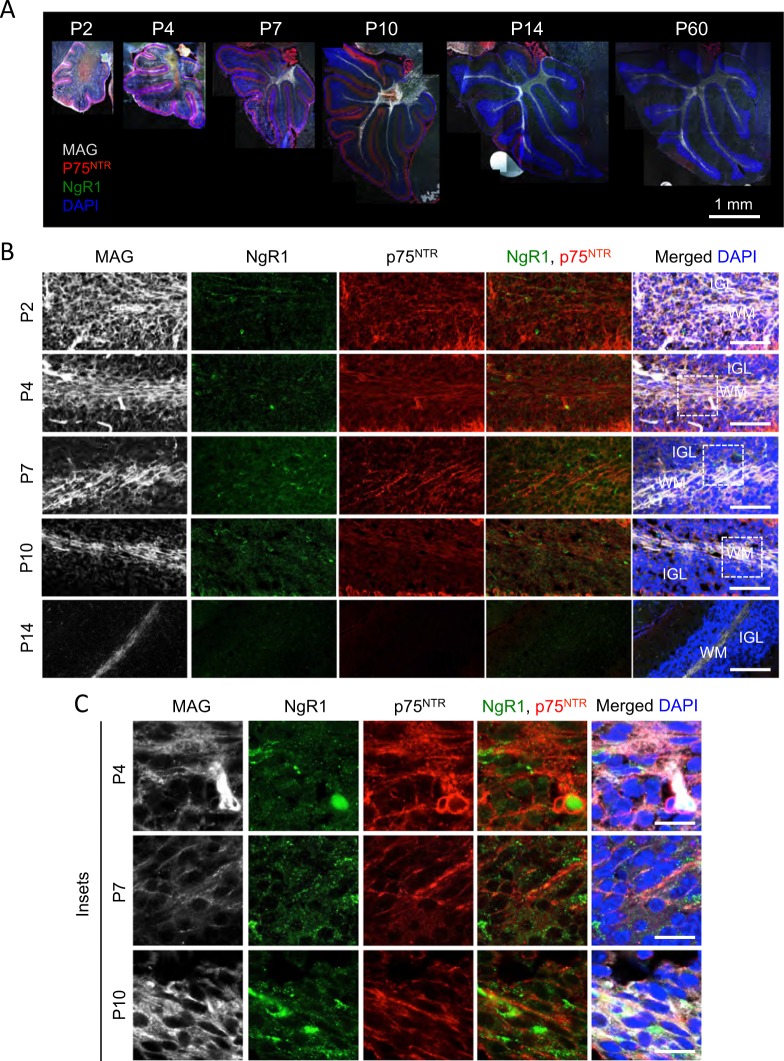
Localisation of MAG, NgR1 and p75^NTR^ in developing cerebellum. **a** Representative images of P2, P4, P7, P10, P14 and P60 wild type whole cerebellar sections triple-labelled by immunoflorescence with anti-MAG, anti-NgR1 and anti-p75^NTR^ antibodies and counterstained with DAPI. Scale bar, 1 mm. **b**, **c** Representative confocal images of P2, P4, P7, P10 and P14 wild-type IGL of folium III triple-labelled by immunoflourescence with anti-MAG, anti-NgR1, anti-p75^NTR^ and DAPI. Scale bar for low magnification images **b** is 50 μm;scale bar inserts **c**, 25 μm. Abbreviations: WM, white matter; IGL, internal granule layer

To establish the role of MAG in developing CGNs, we started the investigation by studying the expression pattern of MAG, NgR1 and p75^NTR^ in CGNs in vivo and in vitro. MAG was expressed in P2, P4, P7, P10, P14 and P60 cerebellar (Fig. 1a–c). MAG immunoreactivity was detected diffusely along the whole P2 and P4 cerebellar, while in P7, P10, P14 and P60 cerebella, it was compartmentalised specifically in the WM (Fig. 1a–c). NgR1 was expressed only in P2, P4, P7 and P10 cerebella (Fig. 1a, b). An intense expression of p75^NTR^ was detected in P2, P4, P7 and P10 cerebella followed by negligible levels in P14 and P60 (Fig. 1a). The highest expression of p75^NTR^ was localised in the Purkinje cell layer (Fig. 1a) in agreement with a previous report^27^. To establish the expression pattern of these molecules in CGNs, we imaged the IGL of folium III from P2 to P14. MAG was expressed in both the IGL and WM at P2 and P4, while was present exclusively in WM at older ages (Fig. 1b, c). Myelinating glia cells that express MAG also express p75^NTR^ ^28–30^. In the folium III IGL, MAG and p75^NTR^ expression overlapped at P2 and P4, while at P7 and P10 the expression of p75^NTR^ was markedly decreased and was undetectable from P14 onwards (Fig. 1b). NgR1 was expressed at P2, P4, P7 and P10, with the highest expression detected at P7, and it was absent from P14 onwards (Fig. 1b). At P2 and P4 the NgR1 positive cells do not express p75^NTR^ (Fig. 1b, c), suggesting that any effects exerted by MAG in CGNs must be in a paracrine manner. All IGL neurons expresses p75^NTR^ at P7 and P10 and a subset of those also co-express NgR1 (Fig. 1b, c).

**Fig. 2:**
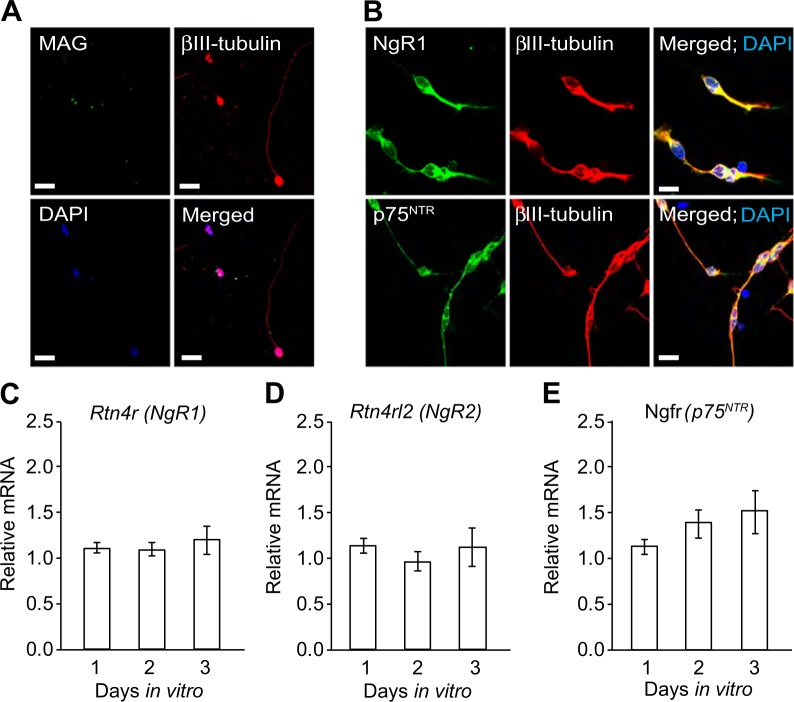
Developing CGNs express NgR1, NgR2 and p75^NTR^ but not MAG. **a–b** Micrographs of representative P7 CGNs double-labelled with anti-MAG (**a**), anti-NgR1 or anti-p75^NTR^ (**b**) combined with anti- β III tubulin and counterstained with DAPI after 24 h in culture. Scale bars, 20 μm. **c–e** *NgR1* mRNA (**c**), *NgR2* mRNA (**d**) and *p75*^*NTR*^ mRNA (**e**) levels relative to the reference mRNA of glyceraldehyde 3-phosphate dehydrogenase (GAPDH) in P7 CGNs cultured for 1, 2 and 3 days in vitro. Mean ± s.e.m of data from eight separate cultures is shown

To verify whether MAG and its receptors are expressed specifically in CGNs, we evaluated their expression in cultured CGNs by immunocytochemistry. As previously observed in vivo, CGNs in culture express NgR1 and p75^NTR^ but not MAG (Fig. 2a, b).

Quantitative PCR (qPCR) analysis revealed NgR1 (Fig. 2c) and p75^NTR^ transcripts (Fig. 2e) in cultured CGNs. Since MAG binds to NgR2^11,16^, we evaluated its expression and found its transcripts in cultured CGNs (Fig. 2d). MAG binds other receptors such as paired immunoglobulin-like receptor B (PirB)^31^, Integrin beta 1 (ITGB1)^32^ and gangliosides (GD1a and GT1b)^8^. qPCR analysis revealed PirB and ITGB1 transcripts in cultured CGNs (Suppl. Fig. 1a-b). We did not examine the presence of gangliosides in those samples because it is not possible to evaluate their expression levels by qPCR, however, since P9 CGNs lysate contains GD1a and GT1b^8^, we expect their presence in P7 CGNs. Upon binding to MAG, these receptors have been suggested to interact with p75^NTR ^^7,8,10,31–33^ making it conceivable that could mediate MAG’s effects in CGNs.

### MAG induces apoptosis through p75^NTR^ in developing CGNs contributing to WM layer refinement

**Fig. 3:**
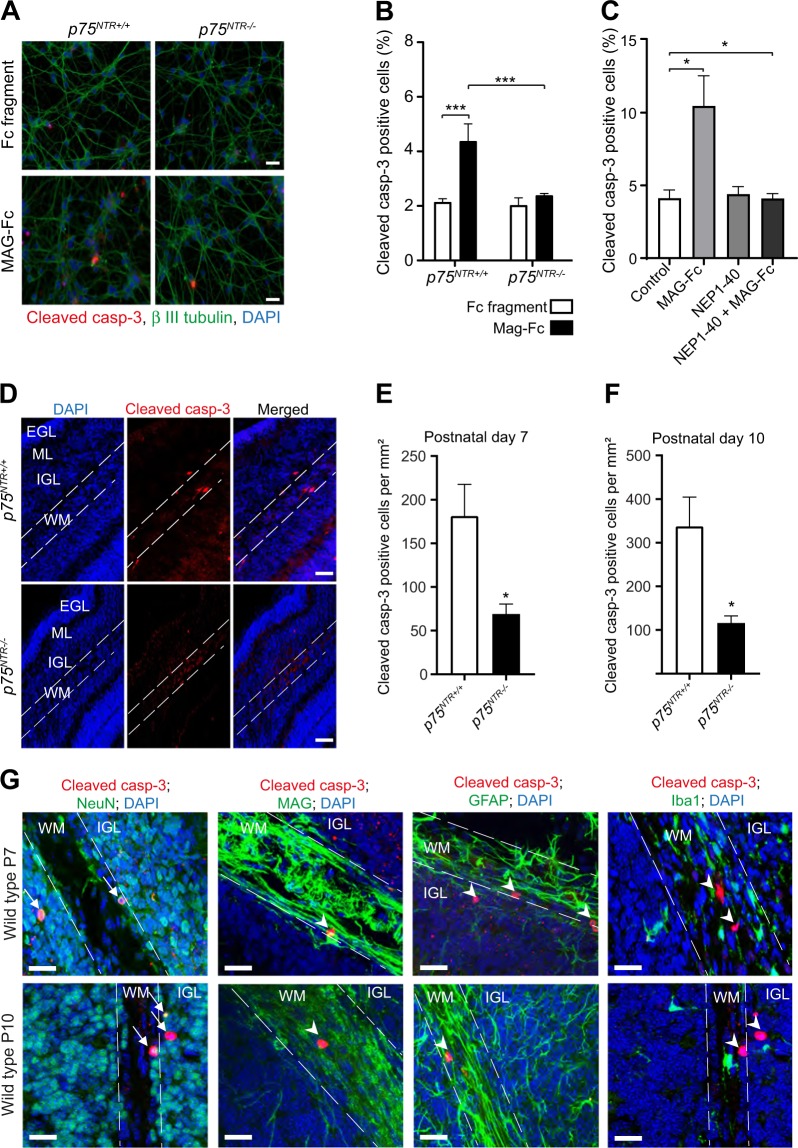
MAG induces cell death in developing CGNs. **a** Representative image of P7 *p75*^*NTR+/+*^ and *p75*^*NTR−/−*^ CGNs cultured for 2 days in vitro prior to treatment with either Fc fragment (control, 25 μg/ml) or MAG-Fc (25 μg/ml) for 24 h. After treatment, cells were stained with anti-cleaved casp-3 (red), anti-β III tubulin (green) and counterstained with DAPI (blue). Scale bars, 20 μm. **b** Percentage of cleaved casp-3 positive neurons in cultured *p75*^*NTR+/+*^ *and p75*^*NTR−/−*^ CGNs treated with Fc fragment (control, 25 μg/ml) or MAG-Fc (25μg/ml) for 24 h (150 images per genotype and condition). Mean ± s.e.m. of data from four separate cultures, ***p < 0.001 compared to control (two-way ANOVA followed by Bonferroni post hoc test). **c** Percentage of cleaved casp-3 positive neurons in cultured wild type CGNs treated with either MAG-Fc (25 μg/ml) alone, NgR antagonist (NEP1-40; 10 μM) alone or MAG-FC plus NEP1-40 for 24 h (40 images per genotype and condition). Mean ± s.e.m. of data from three separate cultures, *p < 0.05 compared to control (one-way ANOVA followed by Bonferroni post hoc test). **d** Representative images of folium III of P7 cerebellum double stained with anti-cleaved casp-3 and DAPI. The outline shows the white matter in folium III. Scale bars, 50 μm. **e–f** Quantification of cleaved casp-3 positive cells in the white matter of folium III from P7 (**e**) and P10 (**f**) *p75*^*NTR+/+*^ *and p75*^*NTR−/*−^cerebella. Mean ± s.e.m, *n* = 6 mice per genotype; *p *<* *0.05*; unpaired Student’s *t* test. **g** Micrographs of folium III of P7 cerebellar sections immunostained for cleaved casp-3 (red) together with either anti-NeuN (green), anti-MAG (green), anti-GFAP (green) or anti-Iba1 (green) and counterstained with DAPI (blue). Arrows shows neurons double-positive for cleaved casp-3 and NeuN while arrow heads indicates neurons positive for cleaved casp-3 that are negative for MAG, GFAP or Iba1. The outlines show the white matter. Scale bars, 50 μm

MAG inhibits neurite growth in different cell types by inducing growth cone collapse through p75^NTR^ ^5,6,10,27^. However, whether MAG also facilitates cell death in CGNs, that is crucial in shaping the cerebellar architecture, has not been explored. Since p75^NTR^ is a death receptor, we asked whether MAG induces cell death in developing CGNs and if such effect is mediated by p75^NTR^. We first examined the effect of MAG on wild type (*p75*^*NTR+/+*^) CGNs in vitro using both propidium iodide (PI) incorporation and cleaved caspase 3 immunoreactivity in the same cultures treated with either Fc fragment (control) or MAG-fusion protein (MAG-Fc) for 24 h. PI stains both necrotic and apoptotic cells, while cleaved caspase 3 will only label apoptotic cells. We observed an increase in PI incorporation in MAG-Fc treated neurons compared to control (Supplementary Fig 2a,b). Similar to PI results, there was an increase in cleaved caspase 3 positive neurons in cultures treated with MAG-Fc compared to control (Supplementary Fig 2a,c). Although, the fold increase in cell death between PI and cleaved caspase 3 positive cells was similar, the number of PI positive cells in control cultures was higher suggesting that some of the cells died independently of MAG-Fc treatment. We therefore, used cleaved caspase 3 staining for subsequent experiments. Next, we investigated the involvement of p75^NTR^ in CGN death. As expected from previous observations (Supplementary Fig 2a,b), *p75*^*NTR+/+*^ CGNs treated with MAG-Fc for 24 h had increased cleaved caspase 3 positive neurons compared to control (Fig. 3a, b). Interestingly, p75^NTR^ knockout (*p75*^*NTR−/−*^) neurons did not respond to MAG-Fc (Fig. 3a, b). MAG exerts its effect by binding to receptors such as NgR1, PirB, β1-integrin and gangliosides, which in turn recruit p75^NTR^ to transduce MAG signal^7,15,33,34^. To determine which of these receptors play a role in MAG-induced cell death of CGNs, we treated *p75*^*NTR+/+*^ CGNs with either MAG-Fc, competitive antagonist of NgR1 (NEP1-40) or MAG-fc plus NEP1-40 for 24 h. As expected, MAG-Fc treatment increased the percentage of cleaved caspase 3 positive CGNs (Fig. 3c). NEP1-40 alone had no effect on CGN death (Fig. 3c). However NEP1-40 blocked MAG-Fc induced cell death in *p75*^*NTR+/+*^ CGNs (Fig. 3c) suggesting that MAG-mediated cell death in developing CGNs requires NgR1 and p75^NTR^.

**Fig. 4:**
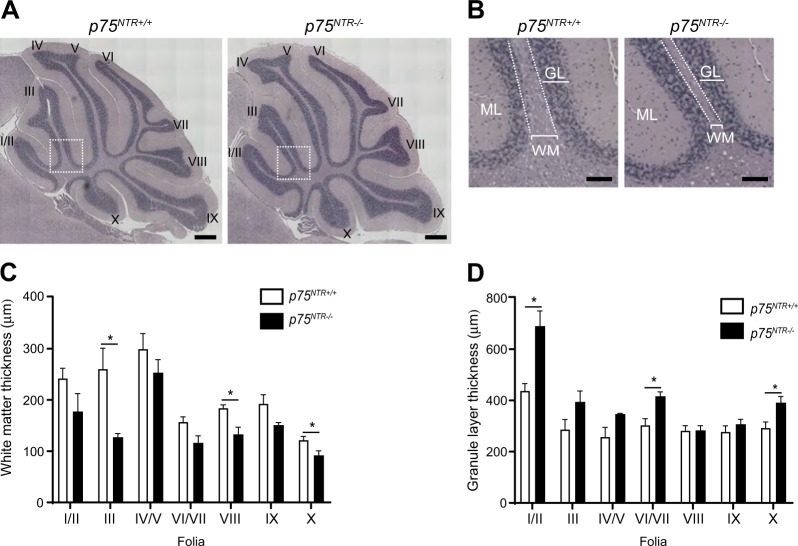
Cerebellar white matter thickness is reduced in adult *p75*^*NTR−/−*^ mice. **a** Hematoxylin and eosin staining of adult (6–9 months) *p75*^*NTR+/+*^ and *p75*^*NTR−/−*^ cerebella. Boxed areas indicate the fields shown in **b**. I/II, III, IV, V, VI, VII, VIII, IX and X indicate the cerebellar folia. Scale bars, 500 µm. **b** High-magnification images of folium III of *p75*^*NTR+/+*^ and *p75*^*NTR−/−*^ cerebella. Examples of WM and GL thickness are highlighted (white brackets). Dotted line delineates the GL/WM boundary. Scale bars, 150 µm. **c**, **d** Quantification of WM (**c**) and GL (**d**) thickness of the different cerebellar folia. The measurements were done at the base of each folia as shown by the white brackets in **b**. Ten cerebellar sections were quantified for each animal. Mean ± s.e.m of data from four *p75*^*NTR+/+*^ and three *p75*^*NTR−/−*^ mice. *p < 0.05 compared to *p75*^*NTR+/+*^ (unpaired Student’s *t*-test). Abbreviations: WM, white matter; GL, granule layer; ML, molecular layer

To assess whether the results obtained in cultured CGNs are relevant for CGN survival in vivo, we counted the number of cleaved caspase 3 positive cells in the WM of P7 and P10 *p75*^*NTR+/+*^ and *p75*^*NTR−/−*^ cerebellar. The number of cleaved caspase 3 positive cells was markedly reduced in *p75*^*NTR−/−*^ cerebellar WM compared to the *p75*^*NTR+/+*^ littermates (Fig. 3d–f), in agreement with a role for MAG/p75^NTR^ signalling in CGN death and WM layer refinement in the developing cerebellum. To confirm that the cleaved caspase 3 cells in the WM were indeed neurons, we stained P7 and P10 *p75*^*NTR+/+*^ cerebellar sections with antibodies against cleaved caspase 3 together with markers for neurons (NeuN), oligodendrocytes (MAG), astrocytes (GFAP) and microglia (Iba1). Cleaved caspase 3 colocalized with NeuN only (Fig. 3g), confirming that these cells are indeed neurons. We then asked whether this CGN death contributes to maintenance of the GL/WM boundary in the adult cerebellum. Consecutive midsagittal adult (6–9-months-old) cerebellar sections stained with hematoxylin and eosin revealed a significant reduction in the WM thickness of folia III, VIII and X in *p75*^*NTR−/−*^ cerebella (Fig. 4a–c) and a trend towards a decrease in the remaining folia (Fig. 4a–c). Conversely, the GL thickness was significantly increased in folia I/II, VII and X and a trend towards an increase was observed in folia III, IV/V (Fig. 4a, b, d). These data suggest that CGN death mediated by MAG/p75^NTR^ signalling contributes to the maintenance of cerebellar GL/WM boundary.

### MAG-induced apoptotic activity in developing CGNs hinders CGN neurite outgrowth

**Fig. 5:**
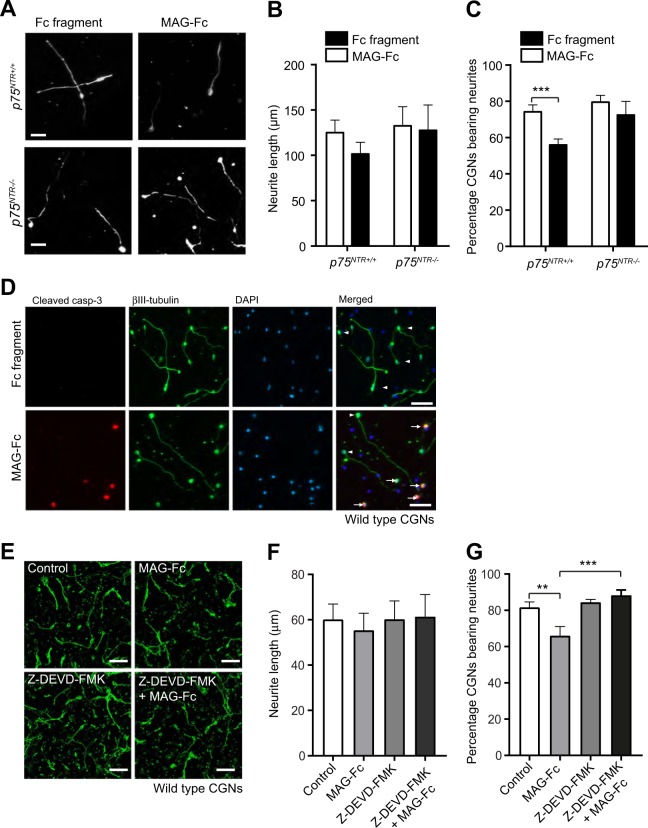
MAG-induced apoptotic activity leads to decreased neurite outgrowth of postnatal developing CGNs. **a–c** Representative images and quantification (**b**, **c**) of P7 *p75*^*NTR+/+*^ and p75^*NTR−/−*^ CGNs cultured for 24 h in medium containing 25 μg/ml Fc fragment (control) or 25 μg/ml MAG-Fc (total of 150 images per genotype and condition). The cells were labelled with anti-β III tubulin. Scale bars, 20 μm. The mean length of the longest neurite (**b**) and the percentage of neurons bearing neurites (**c**) are shown as mean ± s.e.m. of data from three separate cultures, ***p *<* 0.001 compared to control, two-way ANOVA followed by Bonferroni post hoc test. **d** Images of representative wildtype CGNs cultured for 24 h in medium containing 25 μg/ml Fc fragment (control) or 25 μg/ml MAG-Fc (images selected from 60 images per condition). The cells were triple-labelled with anti-cleaved casp-3, anti-β III tubulin and DAPI. Arrows show examples of neurite-less CGNs that are also double-positive for cleaved casp-3 and β III tubulin. Arrowheads show examples of β III tubulin positive and cleaved casp-3 negative CGNs with extended neurites. Scale bars, 50 μm. **e–g** Representative micrographs (**e**) and quantification (**f**, **g**) of wild type CGNs cultured for 24 h in medium containing no factors (control), 25 μg/ml MAG-Fc alone, 10 μM caspase 3 inhibitor (Z-DEVD-FMK) alone or MAG-Fc plus Z-DEVD-FMK. The cells were labelled with anti-β III tubulin. Scale bars, 50 μm. The mean length of the longest neurite (**f**) and the percentage of neurons bearing neurites (**g**) are shown as mean ± s.e.m. of data from three separate cultures (total of 150 images per genotype and condition), **p < 0.01 and ***P < 0.001 compared to control, two-way ANOVA followed by Bonferroni post hoc test

Next, we investigated the relationship between the effects of MAG on CGN death and its reported ability to inhibit axonal growth. We treated CGNs with Fc fragment or MAG-Fc for 24 h, measured the length of the longest neurite and assessed the proportion of neurite-bearing CGNs. The neurite length of *p75*^*NTR+/+*^ and *p75*^*NTR−/−*^ CGNs was similar and MAG-Fc did not alter the length (Fig. 5a, b). However, the percentage of neurite-bearing *p75*^*NTR+/+*^ CGNs was markedly decreased upon MAG-Fc compared to Fc fragment treatment, while percentage of *p75*^*NTR−/−*^ CGNs bearing neurite remain the same after both treatments (Fig. 5a, c). These data suggest that MAG is involved in the initiation of axonal growth of the CGNs but plays no role in the neurite elongation. To ascertain whether this neurite inhibition effect was a consequence of MAG-induced cell death, *p75*^*NTR+/+*^ CGNs were cultured and treated with Fc fragment or MAG-Fc for 24 h. As expected, the percentage of cleaved caspase 3 positive CGNs was increased in MAG-Fc treated neurons (Fig. 5d) compared to control. Surprisingly, the cleaved caspase 3 positive cells had either a short or no neurite, while cleaved caspase 3 negative neurons had neurites with similar length as control cells (Fig. 5d). These data suggest that the MAG-induced neurite inhibition effect in CGNs is a result of MAG-induced apoptosis. To confirm this, we cultured and treated *p75*^*NTR+/+*^ CGNs with the cell-permeant caspase 3-inhibitor, Z-DEVD-FMK 30 min prior to and during MAG-Fc treatment. As expected, *p75*^*NTR+/+*^ CGNs treated only with MAG-Fc showed no difference in neurite length (Fig. 5e, f) but a marked decrease in the percentage of neurons bearing neurites compared to control neurons (Fig. 5e, g). Interestingly, treatment with Z-DEVD-FMK abrogated MAG effect on neurite formation (Fig. 5e, g), emphasising that the reduction in CGNs bearing-neurites induced by MAG is a consequence of increased apoptotic activity in CGNs.

### MAG-induced apoptosis requires p75^NTR^-mediated activation of RhoGDI/cell death pathway

**Fig. 6:**
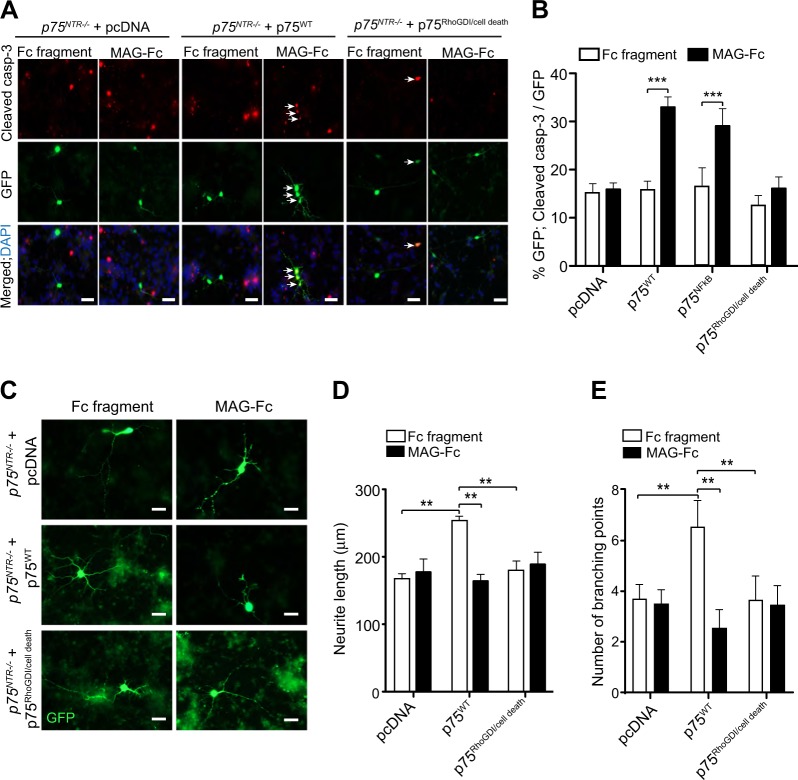
MAG-induced cell death requires coupling of p75^NTR^ to RhoGDI/cell death pathway. **a**, **b** Representative images (**a**) and quantification (**b**) of P7 *p75*^*NTR−/−*^ CGNs transfected on the second day in vitro with either pcDNA (vector), p75^WT^ or p75^RhoGDI/cell death^ constructs. Twenty-four hours after transfection, neurons were treated with either 25 μg/ml Fc fragment or 25 μg/ml MAG-Fc for 24 h and triple-labelled with anti-cleaved casp-3, anti-GFP and DAPI. Arrows indicated neurons double-positive for GFP and cleaved casp-3. Scale bars, 20 μm. The percentage of cleaved casp-3 positive P7 *p75*^*NTR−/−*^ CGNs (**b**) is shown as mean ± s.e.m. of data from four separate cultures, total of 60 images per genotype and condition (***p < 0.001 compared to control, two-way ANOVA followed by Bonferroni post hoc test). **c–e** Representative images (**c**) and quantification (**d**, **e**) of P7 *p75*^*NTR−/−*^ CGNs transfected on the second day in vitro with either pcDNA (vector) or p75^WT^ or p75^RhoGDI/cell death^ constructs and treated 24 h after transfection with either 25 μg/ml Fc fragment or 25 μg/ml MAG-Fc for 24 h. The neurons were labelled with anti-GFP. Scale bars, 20 μm. The mean length of the longest neurite (**d**) and the branch point number (**e**) are shown as mean ± s.e.m. of data from three separate cultures, total of 60 images per genotype and condition (**p < 0.01, compared to control, two-way ANOVA followed by Bonferroni post hoc test)

p75^NTR^ couples to different signalling pathways including NFkB^17–20,27^, JNK/ cell death^21,22^ and RhoGDI^9,14,23–25^ depending on the cellular context^20^. To determine which p75^NTR^ signalling pathway is activated by MAG in developing CGNs, we devised a rescue experiment in *p75*^*NTR−/−*^ neurons transfected with cDNA constructs of wild type p75^NTR^ (p75^WT^) or p75^NTR^ mutants that are selectively deficient in distinct signalling pathways^20,35^. The p75^NFkB^ construct contains the triple mutation D355A/H359A/E363A in the p75^NTR^ death domain that prevents the recruitment of RIP2 thereby hindering p75^NTR^-mediated activation of NFkB^20,27,35,36^. The p75^RhoGDI/cell death^ construct carries the double mutation D410A/S413A in the p75^NTR^ death domain that uncouples p75^NTR^ from RhoGDI and JNK/cell death pathways simultaneously, thereby hindering activation of RhoA and cell death pathways^35,36^. *p75*^*NTR−/−*^ neurons transfected with pcDNA had the same level of apoptosis upon treatment with MAG-Fc or to Fc fragment (Fig. 6a, b). Transfection of p75^WT^ or p75^NFkB^ mutants into *p75*^*NTR−/−*^ CGNs restored the ability of MAG to induce cell death (Fig. 6a, b), indicating that MAG-mediated CGN death is dependent on p75^NTR^ but independent of the NFkB pathway. On the other hand, p75^RhoGDI/cell death^ construct blocked MAG-induced cell death in these neurons (Fig. 6a, b), suggesting that either the RhoGDI or JNK/cell death pathways, or both, are required for MAG-induced cell death in developing CGNs.

To further characterise if these two signalling pathways also play a role in inhibition of neurite formation, we transfected *p75*^*NTR−/−*^ CGNs with pcDNA, p75^WT^ or p75^RhoGDI/cell death^ constructs. Twenty-four hours after transfection, the cells were treated with Fc fragment or MAG-Fc for 24 h. Neurons transfected with p75^WT^ had a marked increase in neurite length and branching compared to pcDNA transfected neurons upon Fc fragment treatment (Fig. 6c–e), indicating a net positive effect of p75^NTR^ overexpression on neurite outgrowth in these neurons. MAG had no effect on neurite length or branching of *p75*^*NTR−/−*^ CGNs transfected with pcDNA but reduced neurite outgrowth in p75^WT^ transfected neurons, although the decrease was not greater than in pcDNA transfected cells (Fig. 6c–e), indicating that the reduction was a result of MAG-induced cell death. Furthermore, CGNs transfected with p75^RhoGDI/cell death^ construct did not respond to MAG-Fc treatment (Fig. 6c–e).

### MAG-mediated CGN death requires activation of the JNK pathway

The inhibition of axonal growth mediated by MAG could be due to activation of RhoGDI/RhoA/ROCK signalling through p75^NTR ^^9,24^, but our data on genetic inactivation of p75^NTR^-mediated RhoGDI/cell death pathways also suggest that the cell death pathway could be involved. Unfortunately, currently there are no p75^NTR^ signalling mutants that can selectively separate those two activities.

**Fig. 7:**
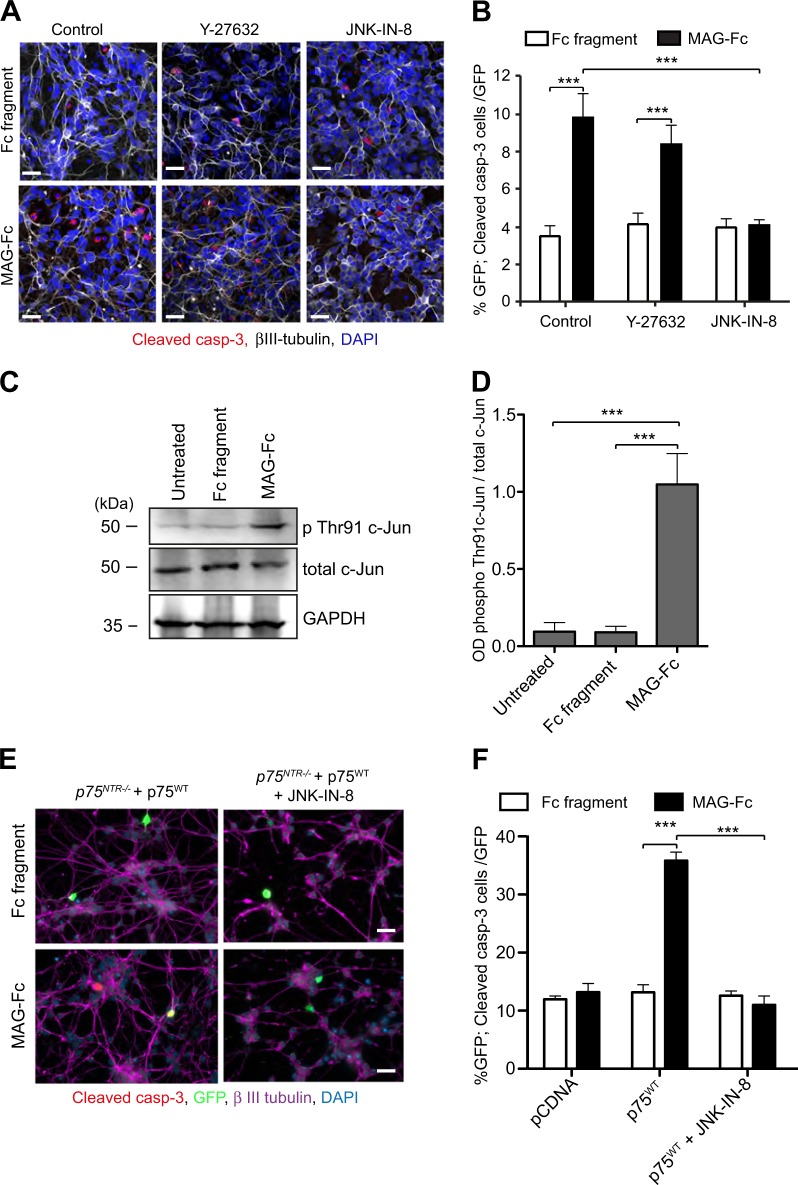
MAG-mediated CGNs death requires activation of the JNK pathway. **a–b** Representative images (**a**) and quantification of the percentage of cleaved casp-3 positive neurons (**b**) of P7 wild type CGNs treated on the second day in vitro with either 25 μg/ml Fc fragment alone, 25 μg/ml MAG-Fc alone, Fc fragment plus 10 μM Y-27632, Fc fragment plus 3 μM JNK-IN-8, MAG-Fc plus Y-27632 or MAG-Fc plus JNK-IN-8 for 24 h and triple-labelled with anti-cleaved Casp-3 (red), anti-β III tubulin (grey) and DAPI (blue). Images in A were chosen from 120 images per condition. Scale bars, 20 μm. Graph in B shows mean ± s.e.m. of data from four separate cultures, total of 120 images per condition (***p < 0.001 compared to control, two-way ANOVA followed by Bonferroni post hoc test). **c**, **d** Representative (**c**) and quantification (**d**) of western blots probed with phospho-c-Jun (Thr91), total c-Jun and GAPDH of lysates from wild type P7 CGNs grown for 24 h prior to 30 min treatment with 25 μg/ml Fc-fragment or 25 μg/ml MAG-Fc. Quantification of c-Jun (Thr91) phosphorylation shown in **d** represent mean ± sem of densitometry from three experiments (***P < 0.001; one-way ANOVA followed by Bonferroni test). **e–f** Representative micrographs (**e**) and quantification (**f**) of P7 *p75*^*NTR−/−*^ CGNs transfected on the second day in vitro with either pcDNA or p75^WT^ constructs and treated 24 h after transfection with either 25 μg/ml Fc fragment alone, 25 μg/ml MAG-Fc alone, Fc fragment plus 3 μM JNK-IN-8 or 25 μg/ml MAG-Fc plus JNK-IN-8 for 24 h. Neurons were quadruple-labelled with anti-cleaved casp-3 (red), anti-GFP (green), anti-MAP2 (magenta) and DAPI (blue). Scale bars, 20 μm. The percentage of cleaved casp-3 positive neurons (**f**) is shown as mean ± s.e.m. of data from three separate cultures, total of 60 images per genotype and condition (***P < 0.001 compared to control, two-way ANOVA followed by Bonferroni post hoc test)

To determine which signalling pathway is mediating MAG-induced cell death in CGNs, *p75*^*NTR+/+*^ neurons were treated with Y-27632, a selective Rho Kinase (ROCK) inhibitor, or JNK-IN-8, a JNK-pathway inhibitor, 30 min prior and during Fc fragment or MAG-Fc treatments. As previously shown, CGNs treated with MAG-Fc alone had increased cleaved caspase 3 immunoreactivity compared to Fc fragment (Fig. 7a–b). Y-27632 did not abrogate MAG-induced apoptosis in CGNs (Fig. 7a, b), while MAG-Fc failed to induce apoptosis in JNK-IN-8 treated neurons (Fig. 7a, b). These data suggest that MAG couples p75^NTR^ to the JNK/cell death signalling pathway to facilitate apoptosis in CGNs.

To verify the activation of JNK signalling pathway, we evaluated phosphorylation of c-Jun on threonine 91 (Thr91) residue, known to be crucial for CGNs apoptosis^27,37^, in *p75*^*NTR+/+*^ CGNs treated with Fc fragment or MAG-Fc. MAG-Fc treatment increased Thr91 c-Jun phosphorylation by six-fold compared to untreated or Fc fragment treated cells (Fig. 7c, d). To further demonstrate the involvement of JNK signalling pathway in MAG-mediated CGNs apoptosis, we transfected *p75*^*NTR−/−*^ CGNs with p75^WT^ construct prior to JNK-IN-8 plus Fc fragment or JNK-IN-8 plus MAG-Fc treatment. Confirming our earlier observations, MAG-Fc induced apoptosis in *p75*^*NTR−/−*^ neurons transfected with p75^WT^ but not pcDNA (Fig. 7e, f), and this effect was blocked by JNK-IN-8 treatment. Collectively, these data indicate that MAG induces cell death in developing CGNs by facilitating p75^NTR^-mediated activation of the JNK/cell death signalling pathway.

## Discussion

MAG, a minor component of myelin in the CNS and PNS inhibits neurite outgrowth and axonal regeneration^4,5,8,10^. Although there is a burgeoning of literature on the role of MAG in developing NS, the effect of MAG has been predominately focused on neurite outgrowth and axonal regeneration^7,8,10^. We show that MAG, its receptor, NgR1, and co-receptor, p75^NTR^, are expressed in the developing cerebellum. We found that from P2 to P4, MAG expression was diffused, while from P7 onwards it was compartmentalised in the WM. Since MAG is reported to be expressed by myelin producing cells^27–30^, we suggest that the expression in developing cerebellum is restricted to oligodendrocytes. We propose that compartmentalisation of MAG is a way to ensure increased MAG protein levels specifically in the cerebellar WM.

NgR1 and p75^NTR^ were expressed from P2 to P10, suggesting that the effect of MAG through NgR1/p75^NTR^ is restricted to these ages. The proportion of CGNs in the IGL at P2 is low but increased markedly with age^1^, arguing that the NgR1 and p75^NTR^ expression might not be on CGNs. Interestingly, the highest levels of colocalization between NgR1 and p75^NTR^ in CGNs were found at P7, the prime point of cerebellar development with the highest levels of CGN apoptotic activity. The absence of MAG in CGNs suggests that it exerts its effect in a paracrine manner to induce apoptosis in the CGNs expressing both NgR1 and p75^NTR^. PirB and ITGB1are expressed by CGNs and therefore they could be also relevant for MAG-induced apoptosis. However, functional data using NgR antagonist proved that NgR1 is required. MAG exert its diverse biological effects through distinct and cell-specific mechanisms^10,34,38,39^. In addition to growth inhibition, MAG protects neurons from acute toxicity^34^, excitotoxicity^40^ and is required for axon regeneration^39^. These and our data highlight the beneficial effect of MAG signalling in the NS and emphasises the diversity of MAG signalling.

In agreement with the in vitro data, we observed increased apoptosis in *p75*^*NTR+/+*^ compared to *p75*^*NTR−/−*^ neurons in the WM that resulted in distorted GL/WM boundary in adult cerebellum. The absence of MAG/NgR1/p75^NTR^ signalling allows CGNs to migrate beyond the GL/WM boundary without consequences until they are stopped by other boundaries such as Purkinje cells axon bundle. We propose that, under physiological conditions, the migrating CGNs that fail to read the stop signal at the cerebellar GL/WM border will enter the WM compartment where they will be exposed to high concentration of MAG. Upon exposure, MAG induces CGNs cytoskeleton collapse and subsequently cell death by similar mechanisms as the ones described for growth cone collapse^41^. In our hands, high concentration (25 μm/ml) of MAG-induced CGN death, while lower concentration had no effect (data not shown). Since oligodendrocytes also express p75^NTR ^^42^, the in vivo effect of MAG on CGNs could be a result of a non-cell autonomous effect. However, our in vitro data where oligodendrocytes are absent, suggest that MAG exerts its effect directly on CGNs.

We propose that MAG is one of the factors involved in the maintenance of cerebellar GL/WM boundary. One report demonstrated that netrin 1 and its receptor UNC5H3 are involved in establishing the GL/WM border by constraining CGNs in the GL^43^. However, it remains unclear what happens to the CGNs that enter the WM compartment. Our study is the first to our knowledge to provide evidence that MAG, through NgR1/p75^NTR^, induces apoptosis of CGNs that fail to read migratory stop cues, thereby refining the WM layer.

Acute MAG/NgR1/p75^NTR^ signalling is essential for growth cone collapse but not for neurite outgrowth inhibition^41–43^. In agreement with that, we show that MAG has no effect in axonal length and neurite elongation in CGNs, but affects initiation of neurite outgrowth. Many neurons failed to initiate neurite outgrowth upon MAG treatment and the majority of these neurons were also apoptotic. Treatment with cleaved caspase 3 inhibitors abrogated MAG effect on neurite formation, suggesting that this phenotype was a result of MAG-induced cell death.

In agreement with previous reports, MAG-induced cell death in *p75*^*NTR+/+*^ but not in *p75*^*NTR−/−*^ CGNs, confirming that MAG’s apoptotic activity requires p75^NTR^ ^12,13,38^. Interaction of MAG with NgR1/p75^NTR^ receptor complex activates the RhoA/ROCK signalling pathway leading to axonal growth inhibition^9,44^, protection of neurons from acute toxicity and excitotoxicity^34,40^ and cell death^45^. One report suggested that MAG, through NgR1/NgR2/p75^NTR^, modulates motor neurons survival after injury in a RhoGDI/RhoA/ROCK signalling-dependent manner^45^. In our study, inhibition of RhoGDI/RhoA/ROCK signalling pathway did not abrogate MAG-induced apoptosis, suggesting that this pathway is not required. On the contrary, our data revealed that MAG-induced apoptosis in CGNs requires p75^NTR^-mediated activation of the JNK/cell death pathway. Both genetic and pharmacological inactivation of p75^NTR^/JNK/cell death pathway hindered MAG-mediated CGN death, while inactivation of RhoGDI/RhoA/ROCK pathway had no effect. Furthermore, MAG induced JNK activity leading to phosphorylation of c-Jun on Thr91. Our data constitutes the first evidence showing that MAG/NgR1/p75^NTR^ complex engages the JNK signalling pathway.

Previously, we reported that p75^NTR^, through the RIP2/NFkB signalling pathway promotes CGN survival upon NGF binding^27^. Pharmacological and genetic manipulations of the RIP2/NFkB pathway leads to CGN death^27^. In the current study, we show that p75^NTR^ can also induce cell death in a subset of CGNs, as a response to a different ligand (MAG) and direct activation of a different intracellular cascade (JNK/cell death pathway) increasing the versatility of this receptor. During cerebellar development most CGNs should survive and arrive to the IGL. However, in physiological conditions a percentage of these neurons should die to remove the excess of neurons and refine cell density in the IGL. Here, we show that the CGNs expressing both NgR1 and p75^NTR^ that carry on migrating past the IGL into the WM layer die through MAG-induced activation of NgR1/p75^NTR^/JNK signalling. The fact that MAG at P7 is already specifically expressed in the cerebellar WM, suggests that this apoptotic mechanism is controlling the elimination of CGNs entering the WM. Since MAG is present in the WM in most areas of the CNS, in the future it will be of interest to investigate whether MAG contributes to WM layer refinement occurs beyond cerebellum.

In summary, we report that MAG induces apoptosis in CGNs by activating the NgR1/p75^NTR^/JNK/cell death signalling pathway, contributing to cerebellar WM layer refinement in developing cerebellum. Our discovery of MAG-mediated apoptosis in developing cerebellum increases our appreciation of the diversity and complexity of MAG signalling in the NS.

## Materials and methods

### Animals

Mice were housed in a 12-h light/dark cycle and fed a standard chow diet. The transgenic mouse line used was p75^NTR^ knockout (*p75*^*NTR−/−*^) mice^46^. *p75*^*NTR−/−*^ mice were maintained in a C57BL/6J background. Mice of both sexes were used for the experiments. All animal experiments were conducted in accordance with the Stockholm North Ethical Committee for Animal Research regulations and the National University of Singapore Institutional Animal Care and Use Committee.

### Immunohistochemistry and immunocytochemistry

For immunohistochemistry, P2, P4, P7, P10, P14 or P60 animals were perfused first with PBS, followed by 4% paraformaldehyde. Harvested brains were post fixed in 4% paraformaldehyde for 16 h and cryoprotected in 30% sucrose before freezing. OCT-embedded brains were frozen at -80 °C overnight and serially sectioned at 10 or 20μm in the sagittal plane using a cryostat. Midline sections were mounted onto electrostatic charged slides (Leica Microsystems), blocked with 5% donkey serum (Fisher scientific) containing 0.3% Triton X-100 (Sigma) in PBS for 1 h at room temperature and then incubated for 16 h at 4 °C with primary antibodies. The sections were washed in PBS before incubated with the appropriate secondary antibodies and counterstained with DAPI (1:10000).

For immunocytochemistry, the cultures were fixed in 4% paraformaldehyde and 4% sucrose for 15 min and washed with PBS before blocking nonspecific binding and permeabilizing with blocking solution (5% donkey serum and 0.3% Triton X-100) in PBS for 1 h at room temperature. Neurons were incubated overnight with the primary antibodies in 1% blocking solution at 4 °C. After washing with PBS, the cultures were incubated with the appropriate secondary antibodies.

The primary antibodies used in this study were: polyclonal anti-p75^NTR^ (Neuromics, GT15057, 1:200), monoclonal anti-MAG (Millipore, Mab1567, 1:200), polyclonal anti-Nogo receptor (NgR1, Alomone, ANT-008, 1:200), polyclonal anti-cleaved caspase 3 (Cell signal, 9761, 1:400), monoclonal anti-NeuN (Millipore, MAB377, 1:200), monoclonal anti-GFAP (Abcam, 1:500), polyclonal anti-Iba1 (Abcam, Ab5076, 1:200), monoclonal anti–β-III tubulin (R&D systems, MAB1195, 1:10000), polyclonal anti-MAP2 (Abcam, ab5392, 1:2000) and polyclonal anti-GFP (Abcam, ab13970, 1:500). Secondary antibodies were Alexa Fluor–conjugated anti-immunoglobulin from Life Technologies, Invitrogen, used at 1:1000 (donkey anti-rabbit IgG Alexa Fluor 555, A31572, donkey anti-goat IgG Alexa Fluor 488, A11055, donkey anti-mouse IgG Alexa Fluor 488, A21202, donkey anti-mouse IgG Alexa Fluor 555, A31570, donkey anti-mouse IgG Alexa Fluor 647, A31571, donkey anti-goat IgG Alexa Fluor 555, A21432 and donkey anti-chicken IgG Alexa Fluor 647, Jackson, 703-496-155). Images were obtained using a Zeiss Axioplan confocal microscope.

### RNA preparation and quantitative PCR

The levels of NgR1, NgR2 and p75^NTR^ mRNAs were quantified by quantitative PCR (qPCR) in total RNA extracted from cultured CGNs relative to a geometric mean of mRNAs encoding the housekeeping enzymes glyceraldehyde phosphate dehydrogenase (GAPDH). Total mRNA was isolated from CGNs cultured for 1–3 days in vitro using the RNeasy Mini Kit (Qiagen) according to the manufacturer’s protocol. cDNA was synthesised by reverse transcription using the High Capacity cDNA reverse transcription kit (Applied biosystems) according to the manufacturer’s protocol. Real-time PCR was conducted using the 7500 Real-Time PCR system (Applied Biosystems) with SYBR Green fluorescent probes using the following conditions: 40 cycles of 95 °C for 15 s, 60 °C for 1 min and 72 °C for 30 s. The following primer pairs were used: NgR1 forward, 5′-TCT GCA GTA CCT CTA CCT ACA A-3′; NgR1 reverse, 5′-GTT GCC ATG CAG AAA GAG ATG-3′; NgR2 forward, 5′-CTG TGG CTC TTC TCC AAC AA-3′; NgR2 reverse, 5′-ACC GAG GTC CAG TTC TTC TA-3′; p75^NTR^ forward, 5′-TAC GTT CTC TGA CGT GGT GA-3′; p75^NTR^ reverse, 5′-GTG TTC TGT TTG TTC TGG CA-3′; PirB forward, 5′-CAA ACT GAG GAT GGA GTG GAG-3′; PirB reverse, 5′-GAC ATG ACA GAA GGT GAG ACA T-3′; ITGB1 forward, 5′-CAG GTG TCG TGT TTG TGA ATG-3′; ITGB1 reverse, 5′-GAT CTG ACC ATT TGA CGC TAG A-3′; GAPDH forward, 5′-ACC ACA GTC CAT GCC ATC AC-3′; GAPDH reverse, 5′-CAC CAC CCT GTT GCT GTA GCC-3′. Forward and reverse primers were used at a concentration of 100 nM each. As a standard for assessment of copy number of PCR products, serial concentrations of each PCR fragment were amplified in the same manner. The amount of cDNA was calculated as the copy numbers in each reverse transcription product and normalised to GAPDH values. Eight separate cultures were analysed for each day.

### Hematoxylin/eosin staining

Adult (6–9 months) *p75*^*NTR+/+*^ and *p75*^*NTR−/−*^ mice were anaesthetised using CO_2_ inhalation, the brains were removed and fixed in 4% paraformaldehyde for 24 h. Brains were dehydrated, paraffin embedded and midsagittal sectioned into 5μm consecutive sections. 10 sections from each animal were deparaffinized using xylene and ethanol and stained with hematoxylin and eosin (H&E) using standard methods.

### Plasmids

Full-length rat p75^NTR^ was expressed from a pcDNA3 vector backbone (Invitrogen). The p75^NFkB^ and p75^RhoGDI/cell death^ mutant constructs have been described previously^35^ and correspond to the triple mutant D355A/H359A/E363A and the double mutant D410A/S413A, respectively. The EGFP plasmid was obtained from Clontech.

### Neuronal cultures

#### Cell death

P7 *p75*^*NTR+/+*^ and *p75*^*NTR−/−*^ CGNs were trypsinized and plated at a density of 40,000 cells per coverslip coated with poly-l-lysine (Sigma, Cat: P7280) in a 24-well plate (Starlab) in basal medium Eagle (BME) supplemented with 10% fetal calf serum (Gibco, Cat: 21010-046), 25 mM KCl (Sigma, Cat: P9541), 1 mM glutamine (Gibco, Cat:25030149) and 2 mg/ml gentamicin (Invitrogen, Cat: 15750060). For assessing cleaved caspase 3, neurons were treated for 24 h starting at the 2 day in vitro. The cells were labelled with cleaved caspase 3 (Cell signal, 9761, 1:400), β-III tubulin (R&D systems, MAB1195, 1:1000) and DAPI. For each experiment and treatment, neurons were cultured in duplicates and at least 15 images were taken per coverslip.

#### Neurite outgrowth

For neurite outgrowth the cells were plated in duplicates at a low density of 5000 cells per coverslip. Treatment started 2 h after plating and lasted for 24 h. Neurons were labelled with β-III tubulin (R&D systems, MAB1195, 1:1000) and DAPI after treatment. 10 images per coverslip were taken using a fluorescence microscope and counted to obtain percentage cleaved caspase 3 over DAPI positive cells.

#### Transfection

For transfection experiments, CGNs were cultured at density of 40 000 cells per coverslip. Neurons were transfected with either pcDNA3, full-length p75^wt^, p75^NFkB^ or p75^RhoGDI/cell death^ plasmids using Lipofectamine LTX kit (Invitrogen, Cat: 15338500) 48 h after plating. 250 ng plasmid per well in the 24-well plate was used.

#### Protein collection

To collect protein for immunoblotting, wild type neurons were cultured at a high density (~200,000 neurons per well) in a 48-well plate. 2 days after plating, neurons were serum, NGF and KCl deprived for 30 min prior to treatment. Cells were then stimulated with either Fc fragment or MAG-Fc for 15 min.

Purified recombinant Fc-fragment (Cat: Ab902285) was purchased from Abcam and MAG-Fc (Cat: 538) was obtained from R&D Systems. ROCK inhibitor (Y-27632, Cat: 72302) was obtained from Stemcell technologies and JNK inhibitor (JNK-IN-8; Cat: 420150) was purchased from Millipore. Caspase inhibitor (Z-DEVD-FMK; Cat 2166) was purchased from Tocris.

### Immunoblotting

Immunoblotting protein samples were prepared for SDS-PAGE in SDS sample buffer (Life Technologies) and boiled at 95 °C for 10 min before electrophoresis on 12% gels. Proteins were transferred to PVDF membranes (Amersham). Membranes were blocked with 5% non-fat milk and incubated with primary antibodies. The following primary antibodies were used at the indicated dilutions: rabbit phospho-c-Jun (Thr91) (Cell signaling, 2303, 1:1000), rabbit anti-c-Jun (Cell signaling, 9165, 1:1000) and rabbit anti-GAPDH (Sigma, G9545, 1:1000). Immunoreactivity was visualised using appropriate HRP-conjugated secondary antibodies. Immunoblots were developed using the ECL Advance Western blotting detection kit (Life Technologies) and exposed to Kodak X-Omat AR films. Image analysis and quantification of band intensities was done with ImageQuant (GE Healthcare).

### Statistical analysis

Data are expressed as mean and standard errors (s.e.m). No statistical methods were used to predetermine sample sizes but our sample sizes are similar to those generally used in the field. Following normality test and homogeneity variance (*F*-test or Kolmogorov-Smirnov test with Dallal-Wilkinson-Lilliefor *p* value), group comparison was made using a unpaired student *t*-test, one-way or two-way ANOVA as appropriate followed by Bonferroni post hoc test for normally distributed data. Mann–Whitney test was done on non-normal distributed data. Differences were considered significant for *p* *<* 0.05. The experiments were not randomised. Data from all experiments are included; none were excluded.

## Supplementary information

Supplementary figure 1

Supplementary figure 2

Supplementary figure legend
