# Correction: MAG induces apoptosis in cerebellar granule neurons through p75^NTR^ demarcating granule layer/white matter boundary

**DOI:** 10.1038/s41419-019-2058-3

**Published:** 2019-10-31

**Authors:** Diana Fernández-Suárez, Favio A. Krapacher, Annika Andersson, Carlos F. Ibáñez, Lilian Kisiswa

**Affiliations:** 1 Department of Neuroscience, Karolinska Institute, S-17177 Stockholm, Sweden; 2 Department of Physiology, National University of Singapore, Singapore, 117597 Singapore; 3 Life Sciences Institute, National University of Singapore, Singapore, 117456 Singapore

**Keywords:** Apoptosis, Cell death in the nervous system, Molecular neuroscience

**Correction to: Cell Death and Disease**

10.1038/s41419-019-1970-x, published online 30 September 2019

The Acknowledgements section in this article is incomplete, and should read as follows:

“We thank Ket Yin Goh and Eunice Sim Weiling for technical support. This research was supported by grants to C.F.I. from Swedish Research Council Vetenskapsrådet, Cancerfonden, European Research Council, and National University of Singapore and to L.K. from Karolinska Institute Research Foundation and Magnus Bergwall Foundation.”

The Author contributions section in also incomplete, and should read as follows:

“D.F.S. conducted and analysed expression pattern. F.K. and L.K. conducted and analysed pharmacological experiments. A.A. did genotyping of the mice. C.F.I. contributed with ideas, advice and provided funding. L.K. designed, conducted and analysed the majority of the experiments and wrote the manuscript with input from all authors.”

