# Supplementary figures and images for: MAG induces apoptosis in cerebellar granule neurons through p75NTR demarcating granule layer/white matter boundary
Source: Cell Death Dis. 2019 Sep 30;10(10):732. doi: 10.1038/s41419-019-1970-x (PMC6768859; doi:10.1038/s41419-019-1970-x)

# Supplementary Figure 1

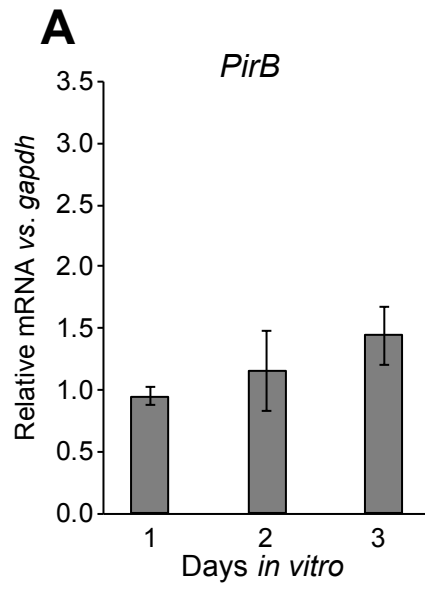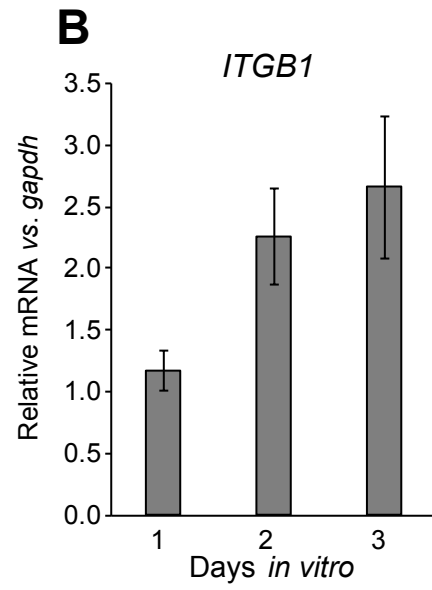

Supplement: Supplementary file 1 — Supplementary figure 1 [file 41419_2019_1970_MOESM1_ESM.pdf]

## Supplementary Figure 2

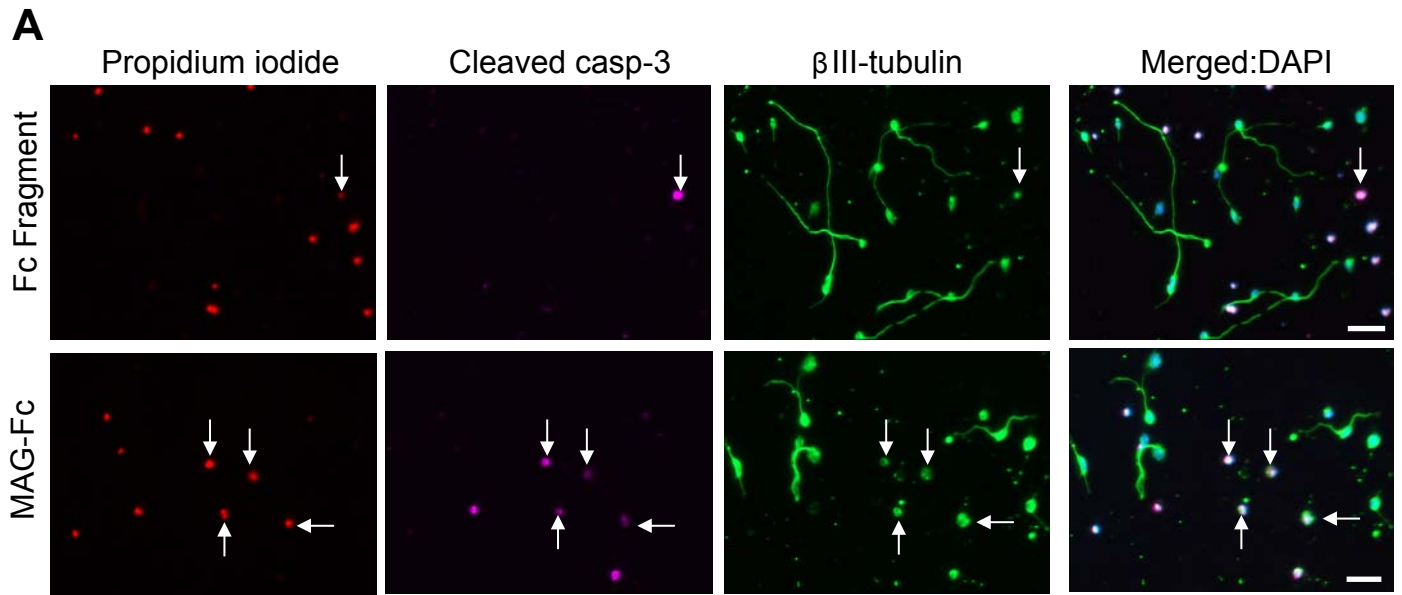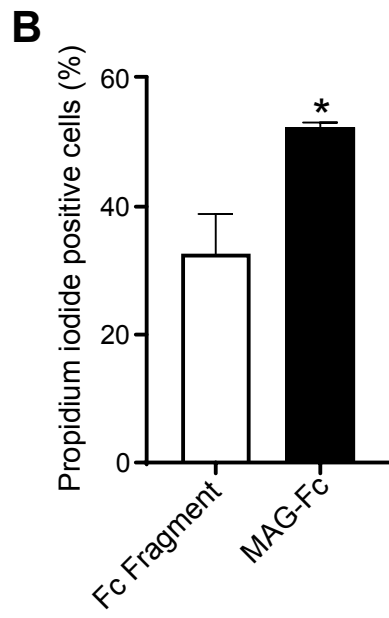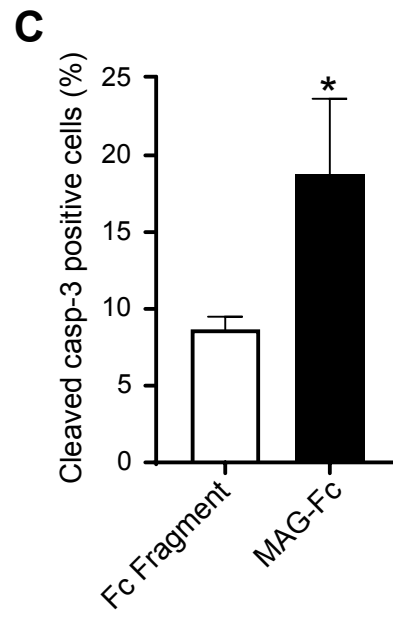

Supplement: Supplementary file 2 — Supplementary figure 2 [file 41419_2019_1970_MOESM2_ESM.pdf]
