# Supplementary material for: MAG induces apoptosis in cerebellar granule neurons through p75NTR demarcating granule layer/white matter boundary
Source: Cell Death Dis. 2019 Sep 30;10(10):732. doi: 10.1038/s41419-019-1970-x (PMC6768859; doi:10.1038/s41419-019-1970-x)
Supplement: Supplementary file 3 — Supplementary figure legend [file 41419_2019_1970_MOESM3_ESM.docx]

**Figure S1. Expression of other MAG receptors in CGNs. Related to figure 2.**

(**A-B**) relative expression of *PirB* mRNA (**A**) and *ITGB1* mRNA (**B**) to the reference mRNA of glyceraldehyde 2-phosphate dehydrogenase (GAPDH) mRNA in CGNs from P7 C57BL6/J animals cultured for 1, 2 or 3 days *in vitro*. Mean ± SEM of data from 8 separate cultures.

**Figure S2. MAG-Fc increases propidium iodide positive cells in culture. Related to figure 3.**

(**A**) Images of representative wildtype CGNs cultured for 24 h in medium containing 25μg/ml Fc fragment (control) or 25μg/ml MAG-Fc. The cells were quadruple-labelled with anti-cleaved casp-3, anti-β III tubulin, propidium iodide and DAPI. Arrows show examples of CGNs triple-positive for propidium iodide, cleaved casp-3 and β III tubulin. Scale bars, 50 μm. (**B-C**) Percentage of propidium iodide (**B**) and cleaved casp-3 (**C**) positive neurons cultured for 24 h in medium containing 25μg/ml Fc fragment (control) or 25μg/ml MAG-Fc. Mean ± s.e.m. of data from three separate cultures, total of 60 images per genotype and condition (**P* < 0.05; unpaired Student’s t-test) is shown.
